# Antioxidants and Phytohormones Act in Coordination to Regulate Sage Response to Long Term Ozone Exposure

**DOI:** 10.3390/plants11070904

**Published:** 2022-03-28

**Authors:** Alessandra Marchica, Lorenzo Cotrozzi, Giacomo Lorenzini, Cristina Nali, Elisa Pellegrini

**Affiliations:** 1 Department of Agriculture, Food and Environment, University of Pisa, Via del Borghetto 80, 56124 Pisa, Italy; alessandra.marchica@agr.unipi.it (A.M.); giacomo.lorenzini@unipi.it (G.L.); cristina.nali@unipi.it (C.N.); elisa.pellegrini@unipi.it (E.P.); 2 CIRSEC, Centre for Climate Change Impact, University of Pisa, Via del Borghetto 80, 56124 Pisa, Italy; 3 Nutrafood Research Center, University of Pisa, Via del Borghetto 50, 56124 Pisa, Italy

**Keywords:** abscisic acid, aromatic herb, ascorbate-glutathione cycle, jasmonic acid, lipoic acid, oxidative stress, salicylic acid, *Salvia officinalis*

## Abstract

Antioxidants and phytohormones are hallmarks of abiotic stress responses in plants. Although it is known that they can offer cell protection or accelerate programmed cell death (PCD) depending on the level of stress, the involvement of these metabolites in stress acclimation is still not fully elucidated. Here, we showed the role of antioxidants and phytohormones in *Salvia officinalis* tolerance to long-term ozone (O_3_) exposure (120 ppb for 36 days, 5 h day^−1^). Salicylic acid (SA) content was increased under O_3_ throughout the whole experiment (+150%, as average compared with control), being required to maintain the cellular redox state and potentiate defense responses. This accumulation was induced before the production of ethylene (ET), suggesting that ET was controlled by SA during O_3_ exposure to modulate the magnitude of chlorosis formation and the cell redox balance (by regulating ascorbate and glutathione levels). The synthesis and/or regeneration of these antioxidants did not protect membranes from lipid peroxidation, as demonstrated by the accumulation of malondialdehyde (+23% as average). However, these processes of lipid oxidation did not include the synthesis of the membrane breakdown products, as confirmed by the unchanged values of jasmonic acid, thus indicating that this compound was not involved in the regulation of PCD strategies.

## 1. Introduction

Plants grow in a continuously changing environment, which has favored the evolution of a highly flexible metabolism and development essential for their sessile lifestyle [1]. Plant metabolism must be highly regulated in order to allow effective integration of a broad spectrum of biosynthetic pathways resulting in antioxidant accumulation and redox signaling activation [2]. Rather than involving simple signaling cassettes, emerging concepts indicate that the relationships between redox state and metabolism are subtle and complex. Plants can be considered as reducing–oxidizing systems in which catabolic processes produce energy, and anabolic processes assimilate it [3]. A key feature determining plant adaptive capability is the extent to which oxidative reactions can be closely controlled. If environmental changes are too extreme to allow short-term metabolic controls to maintain fluxes through primary metabolism, the stress-induced damage ensues [4]. In this situation, functional and genic alterations are induced in an attempt to restore redox homeostasis. If these responses are not occurring appropriately, then primary metabolism becomes impaired, oxidative stress becomes even more severe, and cell death and senescence responses are triggered [5]. Oxidative damage is a widespread phenomenon extensively observed in plants exposed to biotic and abiotic stress.

Tropospheric ozone (O_3_) is a major air pollutant that negatively affects many biological activities in living organisms, being a strong oxidant [6,7,8,9]. Although several efforts have been made to decrease the emission of O_3_ precursors, background O_3_ levels in the Northern Hemisphere are estimated to increase from the current 35–50 ppb to 42–84 ppb by the end of the century, with occasional peaks above 200 ppb [10,11]. Ozone enters the plant through open stomata, and in the apoplast, it breaks down into reactive oxygen species (ROS), whose excessive formation injures DNA, proteins, lipids, and carbohydrates, thus causing reduction of photosynthesis and growth, cell dehydration, accelerated leaf senescence, and the appearance of chlorotic/necrotic leaf injuries [6]. Ascorbate (Asc) and glutathione (Glu) are major ROS scavengers [4], but plant antioxidant power is not limited only to these metabolites, as the detoxification process is a complex network including also other enzymatic and non-enzymatic antioxidants [12]. The roles of some of these antioxidants (e.g., superoxide dismutase, catalase, polyphenols and carotenoids) in plant–O_3_ interaction have been largely investigated (e.g., [4,13]), while those of other antioxidants (e.g., lipoic acid) remain understudied. Phytohormones are also crucial in the regulation of plant growth, development and response to biotic and abiotic stresses, including O_3_ [14,15]. However, it is largely known that cellular responses of plants to O_3_ are dose-dependent. Phytohormones (e.g., ethylene (ET), and jasmonic (JA), salicylic (SA) and abscisic (ABA) acids) have been investigated almost exclusively for their signaling roles in single pulse O_3_ studies (considering a pulse of O_3_ typically greater than 150 ppb, e.g., [16,17,18]), so another aspect that still needs to be elucidated regards the involvement of these molecules in stress acclimation or plant tolerance to long-term O_3_ exposure expected in the near future.

A plant species that has shown a good tolerance to O_3_ [19,20,21,22], as well as to other abiotic stresses (e.g., drought and salinity, [23,24]), is *Salvia officinalis* L. (sage, Lamiaceae family). Native to the Middle East and Mediterranean areas, and naturalized throughout the world, it is a major aromatic herb used in the food and pharmaceutical industries because of its important biological activities, including antioxidant and antimicrobial properties, mainly due to its foliar secondary metabolites [25,26]. Our research group has been studying the sage–O_3_ interaction from different angles for about 10 years [19,20,21,22,27]. Among these investigations, Pellegrini et al. [27] carried out a monthly evaluation of the ecophysiological responses and some antioxidant regulators (i.e., β-carotene and polyphenols) in sage exposed to 120 ppb of O_3_ for three consecutive months (5 h day^−1^), and showed that although photosynthetic activity was reduced already after 1 month of exposure (i.e., the first time of analysis), sage was able to activate an adaptive survival mechanism to complete its life cycle. This capability was mainly attributed to an accumulation of polyphenols [27], and this interpretation seemed then confirmed by Marchica et al. [22], who reported an increased antioxidant capacity twinned with an enhancement of polyphenols occurring during the first weeks of exposure under the same O_3_ concentration (i.e., 120 ppb, 5 h day^−1^) but for only 36 consecutive days. Good O_3_ tolerance and increased antioxidant capacity were also reported by Marchica et al. [19] in sage exposed to a single pulse of O_3_ (200 ppb for 5 h), here highlighting also a key antioxidant role of Asc and Glu. Following the same experimental design, Marchica et al. [20] also reported a crucial signaling network including ROS and phytohormones (i.e., ET, JA, SA and ABA) activated by sage in response to a single pulse of O_3_ exposure. Overall, these researches have led to some very interesting results, but they have also opened up other equally interesting but still unanswered questions.

In the present study, sage was again exposed to 120 ppb of O_3_ for 36 consecutive days (5 h day^−1^) in order to (i) evaluate the effects of long-term O_3_ on sage during the first weeks of exposure, and (ii) characterize the roles of antioxidants (both the most and less investigated) and phytohormones in sage tolerance to long-term O_3_ exposure.

## 2. Results

### 2.1. Leaf Symptoms and Chlorophyll Content

**Figure 1:**
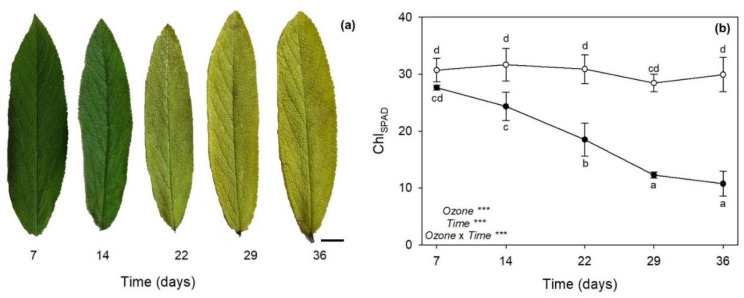
(**a**) Leaf symptoms of *Salvia officinalis* exposed to 120 ppb of ozone (5 h day^−^^1^) for 7, 14, 22, 29 and 36 consecutive days. Bar: 1 cm. (**b**) Variation in relative chlorophyll content (determined in SPAD values, Chl_SPAD_) in leaves of sage exposed to charcoal-filtered air (open circle) or to 120 ppb of ozone (5 h day^−^^1^) for 36 consecutive days (closed circle). Data are shown as mean ± standard deviation. *p* levels for the effects of ozone, time, and their interaction from a one-way repeated measures ANOVA are shown (***: *p* ≤ 0.001). According to Tukey’s HSD post hoc test, different letters indicate significant differences among means (*p* ≤ 0.05).

Ozone-treated plants showed a progressive leaf chlorosis from 14 days from the beginning of exposure (FBE) up to the experimental period (Figure 1a). No symptoms were observed on plants exposed to charcoal-filtered air (i.e., controls), throughout the whole experiment. The one-way repeated measures analysis of variance (ANOVA) of Chl_SPAD_ values showed that the effects of O_3_, time, and their combination were significant (Figure 1b). Ozone also significantly decreased relative chlorophyll content by 23% at 14 days FBE, by 40% at 22 days FBE, and by approximately 60% at 29 and 36 days FBE (throughout the whole text, percentages of O_3_ effects are calculated in comparison with controls at the related times of analysis; Figure 1b).

### 2.2. Gas Exchange and Chlorophyll a Fluorescence

**Figure 2:**
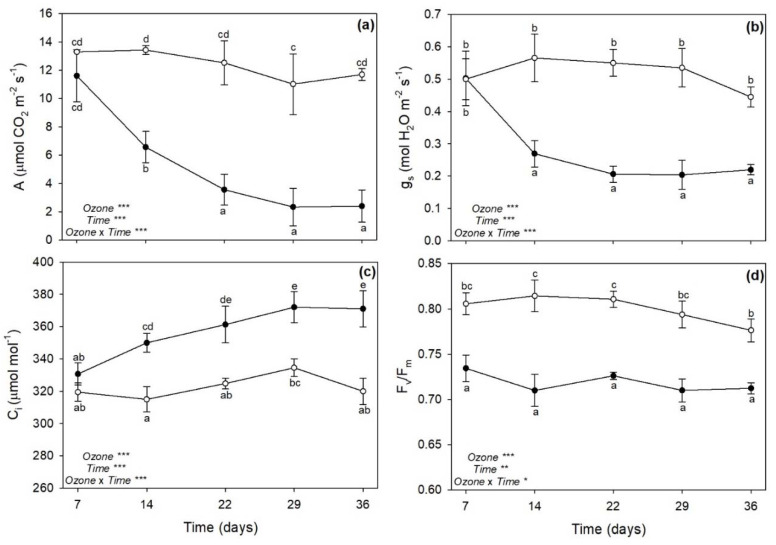
Variation in (**a**) CO_2_ assimilation rate (A), (**b**) stomatal conductance (g_s_), (**c**) intercellular CO_2_ concentration (C_i_), and (**d**) maximum quantum efficiency of the photosystem II photochemistry (F_v_/F_m_) in leaves of sage exposed to charcoal-filtered air (open circle) or to 120 ppb of ozone (5 h day^−1^) for 36 consecutive days (closed circle). Data are shown as mean ± standard deviation. For each parameter, *p* levels for the effects of ozone, time, and their interaction from a one-way repeated measures ANOVA are shown (***: *p* ≤ 0.001, **: *p* ≤ 0.01, *: *p* ≤ 0.05). According to Tukey’s HSD post hoc test, different letters indicate significant differences among means (*p* ≤ 0.05).

The one-way repeated measures ANOVA of ecophysiological parameters showed that the effects of O_3_, time, and their combination were significant (Figure 2). Ozone significantly decreased CO_2_ assimilation rate (A) from 14 days FBE (−51%), with great reductions observed at 29 and 36 days FBE (approximately −80%, Figure 2a); whereas stomatal conductance (g_s_) was similarly reduced by O_3_ from 14 days FBE until the end of exposure (approximately −60%, as average, Figure 2b). Conversely to A, intercellular CO_2_ concentration (C_i_) increased under O_3_ exposure from 14 days FBE (+11%), reaching higher values at 29 and 36 days FBE (+13%, Figure 2c). Ozone decreased the maximum quantum efficiency of the photosystem II (PSII) photochemistry (F_v_/F_m_) already at 7 days FBE (−9%), and this effect was almost consistent until the end of exposure (Figure 2d).

### 2.3. Malondialdehyde and Lipoxygenase Activity

**Figure 3:**
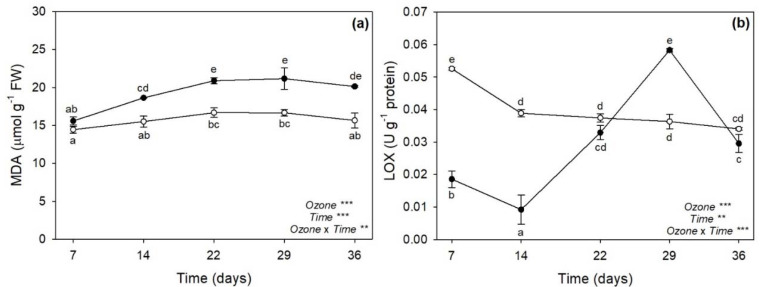
Variation in (**a**) malondialdehyde (MDA) content, and (**b**) lipoxygenase (LOX) activity in leaves of sage exposed to charcoal-filtered air (open circle) or to 120 ppb of ozone (5 h day^−1^) for 36 consecutive days (closed circle). Data are shown as mean ± standard deviation. For each parameter, *p* levels for the effects of ozone, time, and their interaction from a two-way ANOVA are shown (***: *p* ≤ 0.001, **: *p* ≤ 0.01). According to Tukey’s HSD post hoc test, different letters indicate significant differences among means (*p* ≤ 0.05). FW: fresh weight.

The two-way ANOVA of malondialdehyde (MDA) and lipoxygenase (LOX) activity showed that the effects of O_3_, time, and their combination were significant (Figure 3). Ozone significantly increased MDA content by 20% at 14 days FBE and by approximately 25% at later times of analysis (Figure 3a). A variable O_3_ effect was instead reported on LOX activity: it was decreased at 7 and 14 days FBE (−65 and −76%, respectively), did not show differences at 22 days FBE, increased at 29 days FBE (+61%), and came back to control values at the end of exposure (Figure 3b).

### 2.4. Low Molecular Weight Antioxidants

**Figure 4:**
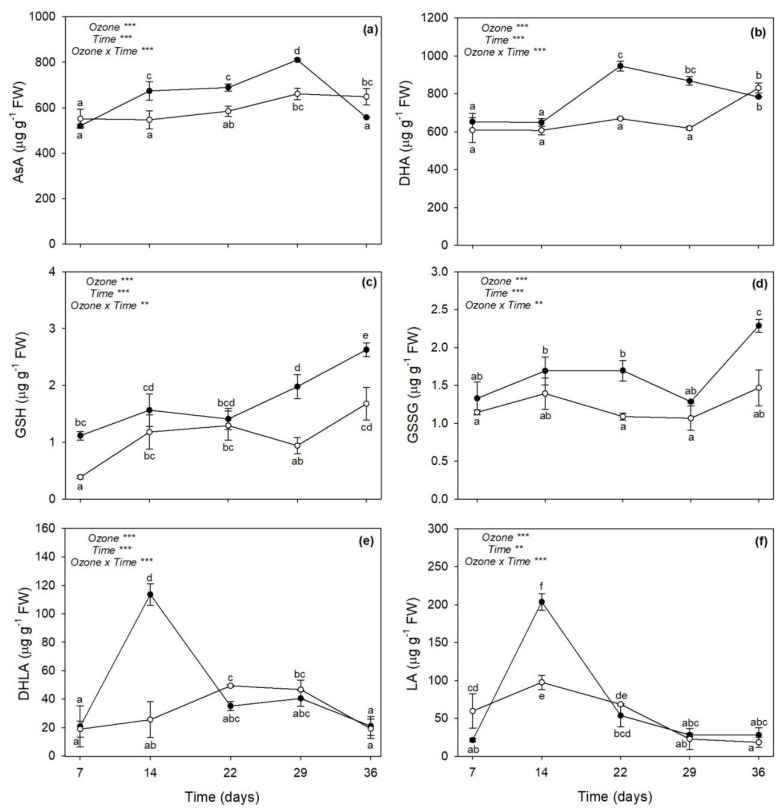
Variation in (**a**) reduced ascorbate (AsA), (**b**) oxidized ascorbate (DHA), (**c**) reduced glutathione (GSH), (**d**) oxidized glutathione (GSSG), (**e**) reduced lipoic acid (DHLA), and (**f**) oxidized lipoic acid (LA) contents in leaves of sage exposed to charcoal-filtered air (open circle) or to 120 ppb of ozone (5 h day^−1^) for 36 consecutive days (closed circle). Data are shown as mean ± standard deviation. *p* levels for the effects of ozone, time, and their interaction from a two-way ANOVA are shown (***: *p* ≤ 0.001, **: *p* ≤ 0.01). According to Tukey’s HSD post hoc test, different letters indicate significant differences among means (*p* ≤ 0.05). FW: fresh weight.

The two-way ANOVA of low molecular weight antioxidants showed that the effects of O_3_, time, and their combination were significant (Figure 4). Ozone significantly increased the reduced form of Asc (AsA) at 14, 22 and 29 days FBE (+22, +21 and +27%, respectively; Figure 4a), as well as the oxidized form (DHA) at 22 and 29 days FBE (around +41%, Figure 4b). Differently, O_3_ increased reduced Glu (GSH) at 7 days FBE (+60%), as well as at 29 days FBE, and even more at 36 days FBE (+63%, Figure 4c). Additionally, oxidized Glu (GSSG) was increased by O_3_ at 22 and again at 36 days FBE (+53 and +56%, respectively; Figure 4d). Ozone also induced a marked accumulation of reduced and oxidized lipoic acid (DHLA and LA, respectively) at 14 days FBE (+109 and +352%, respectively). Conversely, O_3_ reduced LA content at 7 days FBE (−64%). No other significant effects were reported at other times of analysis for these parameters (Figure 4e,f).

### 2.5. Phytohormones

**Figure 5:**
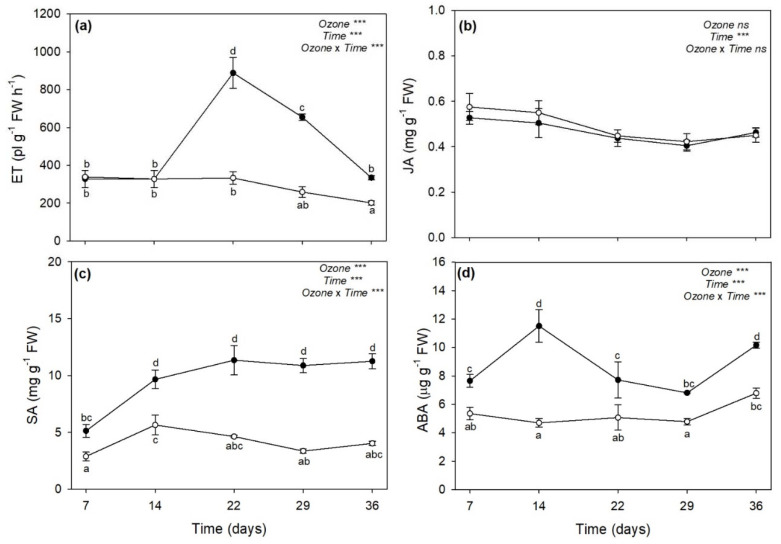
Variation in (**a**) ethylene (ET), (**b**) jasmonic acid (JA), (**c**) total salicylic acid (free plus conjugated forms of SA), and (**d**) abscisic acid (ABA) contents in leaves of sage exposed to charcoal-filtered air (open circle) or to 120 ppb of ozone (5 h day^−1^) for 36 consecutive days (closed circle). Data are shown as mean ± standard deviation. *p* levels for the effects of ozone, time, and their interaction from a two-way ANOVA are shown (***: *p* ≤ 0.001, ns: *p* > 0.05). According to Tukey’s HSD post hoc test, different letters indicate significant differences among means (*p* ≤ 0.05). FW: fresh weight.

The two-way ANOVA of phytohormones showed that the effects of O_3_, time, and their combination were significant (except “ozone” and “ozone × time” for JA; Figure 5). Ozone induced a marked accumulation of ET at 22 days FBE (+166%), which then lowered at 29 (+153%) and 36 (+66%) days FBE (Figure 5a). No O_3_ effects were reported on JA (Figure 5b). Ozone increased total SA (free plus conjugated forms of SA) already at 7 days FBE (+78%), and even more at all the other times of analysis (+150%, as average, Figure 5c). Moreover, ABA increased under O_3_ throughout the whole experiment, peaking at 14 days FBE and again at 36 days FBE (+145 and +49%, respectively; Figure 5d).

## 3. Discussion

As reported in a previous investigation performed by our research group [27], sage plants exposed to 120 ppb of O_3_ (5 h day^−1^) for 90 consecutive days showed leaf yellowing, as well as some disorders in terms of leaf water status and photosynthetic performance, starting from 30 days FBE (i.e., the first time of analysis in that study). As expected, the present study showed that sage plants exposed to the same O_3_ concentration exhibited similar O_3_-induced negative effects already in the first weeks of exposure. Indeed, the severity and incidence of visible injury that develops in plants are commonly utilized as indicators of O_3_ damage [28]; here, a leaf chlorosis was clearly detectable at 14 days FBE, and it developed until the end of exposure. The reader should be aware that a single pulse of O_3_ at a higher concentration (200 ppb for 5 h) did not induce any visible foliar injury in sage [19,20]. The appearance of visible leaf injury because of O_3_ tends to occur by means of two processes. First, O_3_ and O_3_-induced ROS directly harm plant cells by resulting in leaf chlorosis, necrosis and senescence [15]. Second, O_3_-derived ROS work as signal molecules that stimulate the hypersensitive response (HR; [29]). Because senescence and HR are genetically defined cell death programs and show several similarities, it has been speculated that common steps might exist for the induction and/or execution of these two processes [30]. In both cases, phytohormones and signaling molecules play a dual role, either offering protection or increasing/accelerating programmed cell death (PCD), depending on the intensity and duration of O_3_ stress. A single pulse of O_3_ (usually higher than 150 ppb) commonly induces an early synthesis of ET followed by the production of SA, JA, and ABA, where (i) ET and SA signaling triggers ROS production and PCD, establishing a feedback loop, (ii) JA attenuates this cycle by reducing the ROS production, and consequently ET biosynthesis and PCD, and (iii) ABA mainly acts as regulator of stomatal closure and O_3_ influx [15,27]. This crosstalk was also reported in leaves of sage plants exposed to 200 ppb of O_3_ for 5 h [20], but the present study showed a very different regulation of these compounds in sage under long-term O_3_ treatment. In particular, the content of SA was significantly increased by O_3_ throughout the whole period of the experiment, being probably required to maintain cellular redox state and potentiate defense responses [31]. It is known that SA may regulate Glu levels when plants are subjected to various stress factors, by acting as an antioxidant molecule through the Asc-Glu cycle [32]. In the present study, Glu was mainly accumulated at 7 (only the reduced GSH) and 36 days FBE (both GSH and GSSG), whereas both Asc forms were accumulated at 22 and 29 days FBE (likely the crucial stressful period), according to the outcomes reported above. The accumulation of SA was induced before the production of ET, supporting the hypothesis that the level of ET is controlled by SA during O_3_ exposure in order to modulate the magnitude of lesion formation and HR-like cell death by contributing to the regulation of Asc and Glu levels. It should be noted that the synthesis and/or the regeneration of these low molecular weight antioxidant compounds to their reduced powerful antioxidant forms (due to LA after reduction to DHLA as observed at 7 and 14 days FBE) were not able to protect membranes from lipid peroxidation, as demonstrated by the accumulation of MDA (one of the major indicators of cell membrane damage; [33]) from 14 days FBE and even more at the last times of analysis. However, these processes of lipid oxidation did not include the synthesis of the membrane breakdown products formed by LOX, as confirmed by the highly variable response of this enzyme to O_3_ exposure (which decreased at 7 and 14 days FBE, and increased only at 29 days FBE, when most severe symptoms and physiological impairments were first reported) and the unchanged values of JA (thus confirming that this compound was not involved in the regulation of PCD strategies; [34,35,36]). In addition, Marchica et al. [22] documented that the composition of volatile products of LOX pathway (such as C_6_ aldehydes and alcohols and their derivatives) was slightly affected by O_3_ exposure (under the same experimental conditions), suggesting that after a progression of oxidative pressure until 29 days FBE, sage then settled into a stable stress state until the end of exposure (in terms of free fatty acids released by phospholipases from membranes in response to O_3_), confirming a capability of this species to tolerate long-term O_3_ exposure for several weeks.

Our results documented that the production of SA and ET is required to potentiate defense responses in O_3_-treated plants, although leaf injury through the induction of PCD is enhanced starting from 14 days FBE. Symptom observations were in accordance with Chl_SPAD_ values, which confirmed a chlorophyll degradation that followed the same symptom development. A reduction of chlorophylls (i.e., chlorophyll *a* and *b*) as well as of carotenoids (i.e., β-carotene, antheraxanthin, lutein, and violaxanthin) was already reported at 30 days FBE in Pellegrini et al. [27]. Chlorosis is usually associated with alterations of the photosynthetic process [37]. An O_3_-induced reduction of photosynthesis was indeed reported from 14 days FBE, and especially at the last two times of analysis (i.e., 29 and 36 days FBE), thus following the same trend reported for leaf symptoms and Chl_SPAD_. This photosynthetic impairment was clearly due to stomatal limitations, as g_s_ showed reductions similar to those of A (although no higher g_s_ reductions were reported at 29 and 36 days FBE), and to mesophyll limitations, as C_i_ increased following an opposite trend to that of A (i.e., mesophyll cells did not consume CO_2_ for photosynthetic assimilation). The consistent increases in ABA observed throughout the whole experiment lead us to speculate that specific signaling pathways (i.e., xanthophyll and Asc-Glu cycles; [3,38]) are activated in order to modulate stomatal function, photosynthesis, and photoprotection under O_3_ exposure [36,39]. Pellegrini et al. [27] also observed a decrease in A and g_s_ values due to O_3_ at 30 days FBE, but no C_i_ increases were reported in that study (C_i_ decreased). The lack of mesophyll impairment in Pellegrini et al. [27] was likely due to the fact that plants were there exposed to O_3_ in a fumigation facility where temperature, relative humidity and photon flux density were controlled and set at optimal levels for plant growth, thus under better environmental conditions than those of the present study, where plants were exposed to O_3_ in fumigation chambers located inside a greenhouse with natural environmental conditions (except for O_3_ concentration). Indeed, F_v_/F_m_ (i.e., the most widely used photo-oxidative stress marker, [40]) also was not affected by O_3_ in Pellegrini et al. [27], whereas a PSII photodamage was reported at all times of analysis in the present work, thus confirming both the environmental differences between the studies, and more importantly, the harsh O_3_ effects reported in the present study on mesophyll functioning. Finally, it should be noted that the F_v_/F_m_ values were never below 0.70 in O_3_-treated plants throughout the whole period of the experiment, suggesting the idea that F_v_/F_m_ is sensitive not only to O_3_, but also to environmental factors [41].

Overall, the outcomes of the present study suggest that sage was able to orchestrate the regulation of a number of antioxidants and phytohormones, and this capability allowed sage to tolerate long-term O_3_ stress. We speculated that a synergistic interaction among SA, ET and ABA may modulate the defense responses and, at the same time, the magnitude of chlorosis in response to O_3_ exposure. Similar results were previously reported by Guo et al. [35,36] and interpreted as a marker of O_3_-induced leaf senescence [35,36]; this is an aspect that warrants more investigation in order to deeply understand the dual roles of these molecules, as well as the study of these stress-response pathways at a gene expression level. Furthermore, given that increasing O_3_ concentrations and other environmental stresses (e.g., elevated CO_2_ level, drought, and UV) usually occur together, more research would be interesting to further investigate the interactive impacts of multiple environmental stresses on the orchestrated regulations highlighted by the present study.

## 4. Materials and Methods

### 4.1. Plant Material and Experimental Design

Experimental activities were carried out at the field station of San Piero a Grado (Pisa, Italy) run by the Department of Agriculture, Food and Environment of the University of Pisa. At the beginning of spring 2019, 8-month-old seedlings of *S. officinalis* obtained from a local nursery were transplanted in 3.7 L plastic pots with a mix of steam-sterilized soil and peat (1:1, *v*/*v*), and kept under standard conditions until the beginning of the O_3_ exposure. In May 2019, sixty uniformly sized plants (ca. 30 cm tall) were placed in four fumigation chambers inside a greenhouse with natural lighting (the average photon flux density during measurements was around 500 μmol photons m^−2^ s^−1^ at plant height), and kept under charcoal-filtered air (fifteen plants in each chamber). Ten days later, half of the plants at vegetative stage were exposed to a target concentration of 120 ppb of O_3_ (1 ppb = 1.96 µg m^−3^, at 25 °C and 101.325 kPa) for 36 consecutive days (5 h day^−1^, in the form of a square wave from 10:00 a.m. to 03:00 p.m.), while the other plants at vegetative stage were maintained under charcoal-filtered air (control, O_3_ concentration < 5 ppb). Further details about the experimental conditions and methodology are reported in Marchica et al. [22].

Analyses were performed at 7, 14, 22, 29 and 36 days FBE (i.e., approximately weekly). At each time of analysis, at least five plants at vegetative stage per O_3_ treatment (*n* ≥ 5) were randomly selected and measured in terms of gas exchange, chlorophyll *a* fluorescence and relative chlorophyll content (the same plants were repeatedly measured throughout the whole experiment; one leaf per plant). Then, using other plants at vegetative stage (n ≥ 4), five fully expanded leaves (equally distributed over plant height) per plant were excised for ET determination, and another 10 fully expanded leaves (again equally distributed over plant height) per plants were sampled, immediately frozen in liquid nitrogen, and stored at −80 °C until biochemical analyses. All measurements were performed on leaves developed before the beginning of O_3_ treatment.

### 4.2. Gas Exchange and Chlorophyll a Fluorescence

The CO_2_ assimilation rate (A), stomatal conductance (g_s_), and intercellular CO_2_ concentration (C_i_) were determined using an LI-6400 portable photosynthesis system (Li-COR, Lincoln, NE, USA), operating at ambient CO_2_ concentration and saturating light conditions (1500 μmol m^−2^ s^−1^ PAR). Chlorophyll *a* fluorescence measurements were collected using a PAM-2000 fluorometer (Walz, DE). The minimum and maximum fluorescence yields in the dark-adapted state (F_0_ and F_m_, respectively) were determined immediately before and after the application of a saturating light pulse in 40 min dark-adapted leaves (the same used for gas-exchange measurements). The maximum quantum efficiency of PSII photochemistry was calculated as F_v_/F_m_ = (F_m_ − F_0_)/F_m_ [42]. A SPAD 502 m (Minolta, JP) was also used to determine leaf greenness and estimate relative chlorophyll content (Chl_SPAD_). Three measurements per leaf were made, and the mean of these measurements was recorded.

### 4.3. Assessment of Oxidative Damage and Lipoxygenase Activity

Oxidative damage was assessed in terms of lipid peroxidation by measuring the accumulation of malondialdehyde (MDA) by-products [43]. Samples (40 mg fresh weight, FW) were extracted with 1 mL of 80% ethanol. The determination was performed with a fluorescence/absorbance microplate reader (Victor3 1420 Multilabel Counter, Perkin Elmer, Waltham, MA, USA) at 440, 532 and 600 nm.

The lipoxygenase (LOX) activity was assessed by analyzing the oxidation of Fe^2+^ to Fe^3+^ when linoleic acid was added [44]. Samples (150 mg FW) were homogenized with an LOX extraction solution (equal parts of 15 mM CaCl_2_ solution, 13% (*v*/*v*) sucrose, and 60 mM Tris-HCl buffer at pH 6.8). The enzymatic activity was assessed using a UV-Vis 1900 spectrophotometer (Shimadzu, Kyoto, Japan) at 235 nm for 600 s at 50 °C.

### 4.4. Determination of Low Molecular Weight Antioxidant Contents

The total (AsA plus DHA) and reduced ascorbate (AsA) contents were measured as described by Kampfenkel et al. [45], with minor modifications. Samples (60 mg FW) were homogenized with 500 μL of 80% methanol. The supernatant was divided in order to determine the total pool and the reduced form of Asc. The determination was performed using the same spectrophotometer reported before at 525 nm. The oxidized form of Asc was calculated as the difference between total ascorbate and AsA.

The total (GSH plus GSSG) and oxidized glutathione (GSSG) contents were determined according to Sgherri and Navari-Izzo [46]. Samples (50 mg FW) were homogenized with 500 μL of 5% TCA. The supernatant was divided in order to determine the total pool and the oxidized form of Glu. The determination was performed using the same spectrophotometer reported before at 412 nm, for 1 min at 25 °C. The amount of GSH was calculated as the difference between total glutathione and GSSG.

Lipoic and dihydrolipoic acids were extracted by acidic hydrolysis by adding samples (150 mg FW) with 1 mL of 6 N HCl [47]. The supernatants were mixed with 500 μL of chloroform, and the resultant organic fraction was collected and evaporated to dryness under vacuum. Samples were recovered with 500 μL of acetonitrile/0.1% acetic acid in water, 45:55 (*v*/*v*), which was the isocratic mobile phase eluted into an ultra-high-performance liquid chromatograph system (UHPLC; Dionex UltiMate 3000 system, Dionex UVD 170U UV-Vis detector; Thermo Scientific Waltham, MA, USA) equipped with an Acclaim Trinity P1 column (3 µm, 3.0 × 50 mm). The flow rate was 1 mL min^−1^. Both LA and DHLA were detected at 210 nm.

### 4.5. Determination of Phytohormone Contents

A few minutes after excision, ET production was measured by enclosing five leaves from each plant in air-tight containers. After 1 h of incubation at room temperature, gas samples were taken from the headspace of the containers. Separations were performed with a gas chromatograph (HP5890, Hewlett-Packard, Ramsey, MN, USA) equipped with a flame ionization detector and a stainless steel column (150 mm length × 0.4 cm internal diameter, packed with Hysep T). A detailed description of analytical conditions is available in Mensuali-Sodi et al. [48].

Jasmonic acid was determined by using a GC-MS according to Huang et al. [49], with minor modifications. Samples (100 mg FW) were extracted with 1 mL of methanol. The supernatants were evaporated at 37 °C under a vacuum for 10 min, and the residue was re-suspended with 750 μL of ethyl acetate. The extract was injected into an Agilent 8890B gas chromatograph equipped with an Agilent DB-5MS (UI) capillary column (30 m × 0.25 mm; coating thickness 0.25 μm) and an Agilent 5977B single quadrupole mass detector (Agilent Technologies Inc., Santa Clara, CA, USA). A detailed description of analytical conditions is available in Huang et al. [49].

Free and conjugated SA were determined by UHPLC according to Zawoznik et al. [50] with some modifications. Samples (150 mg FW) were extracted with 1 mL of 90% (*v*/*v*) methanol. Separations were performed with the same UHPLC system reported above, equipped with an Acclaim 120 C18 column (5-μm particle size, 4.6-mm internal diameter × 150-mm length) mounted in a Dionex TCC-100 column oven and a Dionex UltiMate 3000 RS fluorescence detector (Thermo Scientific) with excitation and emission at 305 and 407 nm, respectively. The flow rate was 1 mL min^−1^.

Abscisic acid (ABA) content was determined after extracting leaf tissue in 1 mL of distilled water. The determination of ABA was performed at 415 nm with the same fluorescence/absorbance microplate reader reported before using the Phytodetek^®^ Immunoassay Kit for ABA (Agdia, Elkhart, IN, USA).

### 4.6. Statistical Analyses

The normal distribution of data was preliminarily tested by the Shapiro–Wilk test. The effects of O_3_ (between factor), time (within factor), and their combination on Chl_SPAD_, gas-exchange parameters and F_v_/F_m_ were analyzed using a one-way repeated measures analysis of variance (ANOVA). The effects of O_3_, time, and their combination on biochemical parameters were analyzed using a two-way ANOVA. Comparisons among parameter means were determined by the Tukey’s HSD post hoc test. Statistically significant effects were considered for *p* ≤ 0.05. Analyses were performed in JMP 11 (SAS Institute Inc., Cary, NC, USA).

## Data Availability

The data presented in this study are available in the main text.
